# Study on Liver Dysfunction in Type 2 Diabetic Patients in Bangladesh

**DOI:** 10.5005/jp-journals-10018-1155

**Published:** 2016-07-09

**Authors:** Mohd Harun Or Rashid, Md Zahirul Haque, Md Khalilur Rahman, Mohammad Mahbubur Rahman Khan, Abu Shahin Mohammed Mahbubur Rahman, Mamun-Al-Mahtab, Md Salimur Rahman, Projesh Kumar Roy, M Nazrul Islam

**Affiliations:** 1 Department of Hepatology, Rajshahi Medical College, Rajshahi, Bangladesh; 2 Department of Medicine, Rajshahi Medical College, Rajshahi, Bangladesh; 3 Department of Hepatology, Bangabandhu Sheikh Mujib Medical University, Dhaka, Bangladesh; 4 Department of Gastroenterology, Bangabandhu Sheikh Mujib Medical University, Dhaka, Bangladesh; 5 Department of Zoology, Rajshahi University, Rajshahi, Bangladesh

**Keywords:** Abnormal outcome, Diabetes mellitus, Liver functions, Management of DM.

## Abstract

**Aim:**

Diabetes mellitus (DM) represents one of the major lifestyle-related pathological conditions; the incidence and prevalence of DM have reached an epidemic level around the world. Diabetes mellitus is usually associated with obesity, coronary diseases, and cerebral pathologies. However, more insights are required to evaluate a temporal relation between DM and hepatic functions. This study assesses whether and to what extent liver functions are modified in DM patients.

**Materials and methods:**

A total of 100 patients with type 2 DM and 100 normal healthy controls were enrolled in this study following proper scrutiny of inclusion and exclusion criteria. Different parameters of liver function tests were measured in patients in the two groups. Data were analyzed to assess the extent and magnitude of abnormal liver functions in DM.

**Results:**

The levels of bilirubin, alanine aminotransferase (ALT), aspartate aminotransferase (AST), albumin, and prothrombin time were 0.737 ± 0.311 mg/dL, 39.00 ± 24.21 IU/L, 26.42 ± 10.40 IU/L, 4.10 ± 0.513 g/dL, and 16.46 ± 2.78 seconds in patients with DM and 0.506 ± 0.183 mg/dL, 28.26 ± 6.67 IU/L, 18.90 ± 4.75 IU/L, 4.12 ± 0.277 g/dL, and 14.23 ± 1.04 seconds in control subjects. Statistical analyses revealed that most of these parameters of liver function test were significantly different in DM patients compared to control subjects (p < 0.05). Serum alkaline phosphatase level was 89.61 ± 25.59 mg/dL in type 2 DM patients and 96.83 ± 16.34 mg/dL in control subjects (p > 0.05). The prevalence of abnormal values of serum bilirubin, ALT, AST, prothrombin time, and albumin were 5.17, 31.03, 5.17, 5.17, 43.10, and 10.34% respectively in type 2 DM patients and 0, 2, 0, 2, 3, and 0% respectively in control subjects, indicating high prevalence of DM patients with abnormal liver functions.

**Conclusion:**

Abnormal liver functions of different extents and magnitudes have been found in type 2 DM patients, and the impact of abnormal liver function should be considered during the management of DM patients and also to assess their long-term follow-up prognosis.

**How to cite this article:**

Rashid MHO, Haque MZ, Rahman MK, Khan MMR, Rahman ASMM, Al-Mahtab M, Rahman MS, Roy PK, Islam MN. Study on Liver Dysfunction in Type 2 Diabetic Patients in Bangladesh. Euroasian J Hepato-Gastroenterol 2016;6(1):1-4.

## INTRODUCTION

Diabetes mellitus (DM) is a common metabolic disorder characterized by hyperglycemia due to absolute or relative deficiency of insulin.^1,2^ The worldwide prevalence of DM has risen dramatically over the past two decades, from an estimated 30 million cases in 1985 to 177 million in 2000. If the current trend continues, more than 360 million individuals will have DM by the year 2030.^3^ Although the prevalence of both types 1 and 2 DM is increasing worldwide, the prevalence of type 2 DM is raising much more rapidly. Type 2 DM is principally a disease of the middle aged and elderly with typical age of onset > 40 years. The impact of DM is found in both developed and developing countries, and a recent WHO report on diabetes prevalence alarmed that diabetes has posed a serious threat to developing countries with respect to their existing health care delivery service.

Studies have revealed that diabetes, hyperinsulinemia, and coronary risk factors are more prevalent among Bangladeshi migrants compared to native people of the United Kingdom and also compared with other South Asian groups (Indian, Pakistani) settled in the United Kingdom.^4,5^ Also, it has also been reported that Bangladeshis among the entire South Asian immigrants had highest mortality and attack rate from coronary heart disease.^6^

Considering the direct and indirect impacts of DM on the health care delivery system of Bangladesh, there is an emergent need to develop broad insights into the pathogenesis of DM in this country. This may pave the way for containment of DM and DM-induced complications. However, there is paucity of information about characteristic features of DM. In another spectrum, the effects of DM on the heart and brain have been reported to some extent. But the effects of DM on the liver and its functions are still limited; almost nothing is clear about the impact of DM on the liver of Bangladeshi migrants.

This study attempts to clarify the effect of DM on the liver. The study has been designed to compare liver functions in one group of patients with DM in Bangladesh and the other group representing normal control subjects without any liver disease. This study would be helpful to develop insights into the interrelation between DM and liver function, not only in Bangladesh but also in other developing countries harboring comparable socioeconomic conditions like Bangladesh.

## MATERIALS AND METHODS

The study was conducted at Rajshahi Medical College Hospital and Rajshahi Diabetic Association Hospital, Rajshahi, Bangladesh. Consecutive 100 patients with type 2 DM and 100 apparently healthy people were enrolled in this study. The study was conducted from July 2012 to June 2014. The age of the subjects was more than > 40 years. Patients of both sexes were included. The diagnosis of type 2 DM was done based on the WHO criteria. Patients with history of alcoholism and with the habit of using hepatotoxic drugs like acetaminophen, NSAIDS, methotrexate, amiodarone, bleomycin, tamoxifen, sodium valproate, metformin, and pioglitazone were excluded from this study. Patients taking insulin were also discarded from the study. Finally, patients with acute and chronic liver diseases were also not enrolled in this.

All patients had a history of DM. To affirm the diagnosis of DM, blood sugar and HBA1C were measured. The serum levels of bilirubin, alanine aminotransferase (ALT), aspartate aminotransferase (AST), alkaline phosphatase (ALP), and albumin were measured using commercial methodology. Prothrombin time was also measured in all subjects. Hepatitis B surface antigen (HBsAg) and antibody to hepatitis C virus (HCV) were measured in all subjects to discard HBV and HCV infection. Lipid profile of all patients was also estimated to develop insights into the relation between DM and different parameters of lipid metabolism. Abdominal ultrasonography of patients was also accomplished. Informed consent was taken from all patients after explaining the nature and purpose of the study. The study also received ethical clearance from respective institutions.

### Statistical Analysis

Data were analyzed with the help of Statistical Package for the Social Sciences (SPSS) software program and expressed as mean ± SD. p-value < 0.05 was considered significant.

## RESULTS

### Demographic Features of the Study Population

Among patients with DM, 56 (56%) were male and 44 (44%) were female; in the control group, out of 100 subjects, 56 (56%) were male and 44 (44%) were female. The mean age of type 2 DM patients and normal healthy control people were 54.06 and 55.30 years respectively. Among the DM patients, 58 (58%) and 42 (42%) lived in urban and rural areas respectively, and in the control group, 60 (60%) and 40 (40%) lived in urban and rural areas respectively. Among DM patients, 18 (18%), 16 (16%), 30 (30%), and 36 (36%) were farmers, businessmen, service holders, and housewives respectively. On the contrary, in the control group, 16 (16%), 14 (14%), 50 (50%), and 20 (20%) people were farmers, businessmen, service holders, and housewives respectively.

### Abnormal Liver Functions in Patients with Type 2 DM

Blood sugar levels were significantly higher in patients with DM than in control subjects (7.33 ± 2.85 *vs* 5.21 ± 0.52 mg/dL, p < 0.05), as expected. Also, the levels of Hb1AC were significantly higher in DM patients than in controls (7.79 ± 1.68 *vs* 5.69 ± 0.360 mg/dL, p < 0.05).

The levels of serum bilirubin were significantly higher in DM patients (0.73 ± 0.32 mg/dL) compared to normal subjects (0.50 ± 0.18 mg/dL) (p < 0.05); however, the mean value of both group was within the normal range.

Similar trends about higher levels of ALT and AST were found in type 2 DM patients compared to control subjects (Table 1). However, the levels of ALP were not significantly different between DM patients and control subjects (89.61 ± 25.59 *vs* 96.83 ± 16.36 mg/dL, p > 0.05). Prothrombin time of type 2 DM and normal people were 16.46 ± 2.78 and 14.23 ± 1.04 seconds respectively (p > 0.05). The levels of serum albumin were significantly decreased in DM patients compared to control subjects (p < 0.05) (Table 1).

**Table 1:** **Table 1:** Liver function tests results of type 2 DM patients and control group

*Liver function test*		*Type 2 diabetic* * patients (n = 100)* * (mean ± SD)*		*Control group* * (n = 100)* * (mean ± SD)*		*p-value*	
Serum bilirubin		0.7372 ± 0.3118		0.5063 ± 0.1831		< 0.05	
Alanine Aminotransferase		39.00 ± 24.21		28.26 ± 6.67		< 0.001	
Aspartate aminotransferase		26.42 ± 10.40		18.90 ± 4.75		< 0.001	
Serum alkaline phosphatase		89.61 ± 25.59		96.83 ± 16.34		> 0.05	
Prothrombin time		16.46 ± 2.78		14.23 ± 1.04		< 0.01	
Serum albumin		4.10 ± 0.513		4.12 ± 0.277		< 0.05	

Data are presented as mean and ± SD; n: Number of subjects; SD: Standard deviation

### Increased Prevalence of Abnormal Liver Functions in DM Patients

Among the 100 type 2 DM patients, 58 had abnormality in either one or more parameters of liver function test. Three of these patients had raised serum bilirubin (3%), 18 (18%) had raised ALT, 3 (3%) had raised AST, 3 (3%) had raised serum ALP, 25 (25%) had prolonged prothrombin time, and 6 (6%) had decreased serum albumin. On the contrary, among healthy control subjects, none had any abnormal serum bilirubin, ALT, and serum albumin. Only two control subjects had raised ALT, 2 had raised ALP, and 2 had prolonged prothrombin time. Increased levels of ALT and prolonged prothrombin time and decreased serum albumin were highly prevalent in DM patients.

### Severity of Abnormal Liver Functions in DM Patients

Three patients had abnormal serum Bilirubin, and this was mildly elevated in all patients. Eighteen patients had elevated ALT, and among them 12 patients had mildly elevated ALT and 6 had moderately elevated ALT. All patients with elevated AST and ALP had mildly elevated levels of these enzymes. Twenty-five patients had prolonged prothrombin time, of which 17 were mildly prolonged and 8 were moderately prolonged. Among 6 patients with decreased serum albumin, 4 had mildly decreased and 2 had moderately decreased levels of albumin.

### Nutritional Status of the Study Population

Among the 100 type 2 DM patients, 70 subjects had normal BMI (18.5–24.9 kg/m^2^), 20 were overweight (BMI 25.0–29.9 kg/m^2^), 4 were obese (BMI > 30.0 kg/m^2^), and 6 were malnourished (BMI < 18.5 kg/m^2^). Among the 100 normal control subjects, 70 had normal BMI, 10 were overweight, 20 were obese, but there were no malnourished people in this group.

### Abdominal Ultrasonography Findings of DM Patients

Among the 100 patients with type 2 DM, 40 (40%) had normal liver, 34 (34%) had mild fatty changes, and 23 (23%) had moderate fatty changes in the liver. Three patients had mild hepatomegaly. None had cirrhosis or hepatocellular carcinoma.

## DISCUSSION

The study presented here has unmasked some important and relevant information about the impact of DM on the liver. The study was designed to assess the impact of type 2 DM on liver functions, and in fact, separate studies would be required to evaluate the impacts of other types of DM on the liver and its functions. Several investigators have shown that DM is related to lifestyle-related pathological conditions and is associated with coronary disease and cerebral pathologies. Also, an association between nonalcoholic fatty liver diseases (NAFLD) and DM has been shown. Thus, it is plausible to consider whether NAFLD is related to the pathogenesis of DM or NAFLD is a contributing factor for the progression of DM. Truly speaking, the impacts of DM on the liver and its functions have not been adequately analyzed. Also, it is needless to mention that these types of analytic studies have rarely been reported from developing countries like Bangladesh in spite of the fact that the incidence and prevalence of both DM and DM-associated complications are on a rising trend in these countries.

In this study, abnormal liver functions were recorded in 58% patients with type 2 DM and only in 6% normal controls. Thus, DM patients are susceptible to liver damages. It is important to clarify if DM-induced abnormal liver function or abnormal liver functions were predisposing factors for DM. Serial sera samples could not be taken from these patients to provide firm evidence about this, and also we are unaware of the time point at which DM started and also when abnormal liver functions precipitated in these patients. However, circumstantial evidence implies abnormal liver functions may have followed DM and DM may be responsible for abnormal liver functions. The patients with DM were not suffering from any comorbidity that can cause liver damages, such as virus, drug, or other pathologies of liver damages. However, liver damages in DM patients may be induced or due to prevailing NAFLD. To elucidate these facts, we also checked BMI and hepatic fat in these patients. The BMI of DM patients and control subjects were almost comparable. Paradoxically, obesity was more prevalent among control subjects than DM patients. Thus, accumulation of fat and obesity cannot be accounted for impaired liver functions of DM patients in this series.

Salmela et al showed that 17.0% patients with DM in their study had abnormal ALT.^7^ In a larger study, Erbey et al showed elevated ALT in 7.8% patients with DM compared to 3.8% patients without DM.^8^ Gonem et al have shown increased ALT, ALP, and bilirubin in 15.7, 10.4, and 37% patients with type 2 DM. In this study, elevated ALT and serum bilirubin were detected in 18 and 3 patients with DM respectively.^9^ Thus, more studies from different geographical regions would be warranted to develop proper insights into the prevalence of abnormal liver functions in DM patients.

Cusi and Kenneth in 2009 showed that approximately 70% person with type 2 DM had a fatty liver and the disease follows a more aggressive course with necroinflammation and fibrosis. New evidence suggests that it is not steatosis per se but the development of lipotoxicity-induced mitochondrial dysfunction and activation of inflammatory pathways that leads to progressive liver damage.^10^ Nonalcoholic steatohepatitis is a leading cause of end-stage liver disease. In our study fatty changes in the liver was seen in 57% patients, among whom 34% had mild fatty changes and 23% had moderate fatty changes. Thus, exploration of the degree of fatty changes in DM patients and proper management of these patients would restrict progression to several liver-related pathologies.

Sidhartha et al^6^ showed that about one-fourth of type 2 DM patients had a body mass index (BMI) below 19, i.e., low body weight type 2 DM in India. In our study, 6% of type 2 DM patients were malnourished, i.e., had BMI < 18.5 (kg/m^2^) and 70% were normal, i.e., had BMI within the range 18.5 to 24.5. Thus, in addition to a relation of increased BMI in DM, low body weight patients with DM should be considered for proper management of this group.

Taken together, this study has shown increased prevalence of abnormal liver functions in patients with type 2 DM compared to control subjects. Although this study is endowed with considerable limitation of small sample size and single-point assessment of liver functions, it may act as an eye opener. More studies of this nature should be conducted in developing countries to get proper insights into the involvement of liver in DM and also to determine the proper design of management of these patients.
